# Perioperative immunonutrition intervention on postoperative outcomes among gynecological cancer patients under enhanced recovery after surgery setting: A study protocol of explanatory mixed method study

**DOI:** 10.1371/journal.pone.0315568

**Published:** 2024-12-31

**Authors:** ChiouYi Ho, Zulfitri Azuan Mat Daud, Barakatun Nisak Mohd Yusof, Hazreen Abdul Majid

**Affiliations:** 1 Department of Dietetics, Faculty of Medicine and Health Sciences, Universiti Putra Malaysia, Serdang, Selangor, Malaysia; 2 Department of Dietetics and Food Service, Institut Kanser Negara, Ministry of Health Malaysia, Wilayah Persekutuan Putrajaya, Malaysia; 3 Research Centre of Excellent on Nutrition and Non-Communicable Diseases (RCoE-NNCD), Universiti Putra Malaysia, Serdang, Selangor, Malaysia; 4 School of Health Rehabilitation Sciences, Health Sciences University, Bournemouth, United Kingdom; 5 Department of Social and Preventive Medicine, Faculty of Medicine, University of Malaya, Kuala Lumpur, Malaysia; University of Ilorin, NIGERIA

## Abstract

**Background & aims:**

Enhanced Recovery After Surgery **(**ERAS) has shown significant improvements in postoperative outcomes and a reduction in complications, while immunonutrition (IMN) has been shown to modulate the immune system and inflammatory response. However, many studies have overlooked the crucial aspects of nutrition status and patient perception within the intervention approach. This study aims to investigate the efficacy and explore patients’ acceptance of the IMN intervention in postoperative outcomes among gynecological cancer (GC) patients under the ERAS framework.

**Methods:**

This two-phase explanatory sequential mixed-method study design comprises an open-labeled randomized control trial and a qualitative study. The GC participants will be randomly allocated into intervention and control groups. Malaysian adults scheduled for elective surgery will be recruited, with the intervention group receiving IMN for five days before and seven days after elective surgery, while the control group undergoes routine nutritional care before the operation. Both groups will adhere to the ERAS protocol. An explanatory qualitative study will be conducted among GC patients to elucidate their expectations following the trial. Study outcomes include hospitalization duration, change in nutrition status, biochemical profile, functional status, and quality of life. Additionally, the secondary outcome focuses on evaluating the perception of the intervention approach. Quantitative and qualitative data will be analyzed on an intention-to-treat basis and through inductive thematic analysis, respectively.

**Conclusion:**

Implementing perioperative IMN intervention within the ERAS framework may contribute to the preservation of better nutrition status and the provision of sufficient dietary intake to support postoperative recovery, and promote better surgical outcomes. Patients’ perceptions play a pivotal role in enhancing understanding of disease management and adherence to the intervention approach.

**Trial registration:**

NCT06039306.

## Introduction

Surgery serves as an effective treatment for gynecologic oncology (GC) patients [1]. However, surgery induces systemic inflammatory response syndrome and a multifactorial immunosuppressive catabolic condition characterized by proteolysis, weight loss, and other symptoms [2]. Hence, the Enhanced Recovery After Surgery (ERAS) program aims to mitigate issues, alleviate patient stress from associated procedures, reduce the length of hospital stay, and enhance postoperative recovery [3, 4]. Active nutritional support can improve treatment efficacy and decrease the likelihood of complications. As a vital component of the ERAS process, Oral Nutrition Support (ONS) is a crucial component of the system [5].

Post-operative nutrition management is crucial to preserve muscle mass and maintain quality of life [6]. Hydroxymethylbutyrate (HMB) has been proven to preserve muscle mass and promote muscle production [6]. Immununutrition (IMN) ONS is particularly recommended to modulate the immunological response [7]. IMN intervention is expected to restore the appropriate metabolic and immunological response [8]. The IMN nutrients, including omega-3 fatty acids, nucleotides, glutamine, arginine, and trace minerals (iron, zinc, copper, and selenium), have been studied for their role in immunomodulating diets in wound healing in recent years [9]. These immunonutrients aim to minimize tissue injury, lower the risk of cancer development, and also affect cellular defenses, local or systemic inflammation, and mucosal barrier function [10, 11].

Systemic review summarized the impact of IMN post-operative clinical results including length of stays, infection rate, and postoperative complication in gastrointestinal surgery [12]. Even though it has mainly been used in the immediately following surgery, the early study also mentioned its usage as an ONS before the esophagectomy surgery (3–5 days before surgery) [12]. However, there is still no solid evidence regarding the efficacy of IMN intervention on postoperative outcomes among patients with gynecological cancer in Southeast Asia’s ERAS environment [13]. Therefore, the research gap on the impact of IMN intervention on postoperative outcomes among gynecological cancer patients under the ERAS scenario in surgical GC patients must be addressed. The significance of perioperative nutrition in influencing the patient’s metabolic status, nutritional status, and physiological well-being underscores the need to provide appropriate energy and substrates to facilitate recovery. Achieving this often requires a multimodal strategy, involving education, training, and behavior modification strategies, to effectively translate evidence-based recommendations into reality [14]. Before initiating any changes, it is crucial to comprehend the perspectives of those involved and the environment in which these changes will occur [15]. Patients’ perceptions play a key role in the execution of postoperative nutrition and the success of postoperative nutrition intervention plans.

Our knowledge of the discrepancies between factors influence or barriers that have not been investigated before among patients with gynecological cancer might be improved by the currently proposed qualitative study. The qualitative comparative analysis will be applied to explore agreement between qualitative syntheses of data on patients’ views and evidence from trialed interventions to increase adherence to treatments [16]. This study aims to shed light on the importance of incorporating patients’ perspectives for a better understanding of disease management. The potential value of using qualitative research is to triangulate the study findings. [16]. Consequently, developing focused initiatives to enhance perioperative feeding practices among patients with gynecologic cancer will entail developing a better theoretical understanding of patients’ perceptions. The explanatory qualitative study aims to explore the perception of GC patients and the disparity in the effectiveness of perioperative nutrition intervention.

## Method and materials

This study adopts a two-phase explanatory sequential mixed method design, encompassing an open-labeled randomized control trial and followed by a qualitative study. The research will be conducted at Institut Kanser Negara. This study will focus on the effectiveness of perioperative IMN intervention on postoperative outcomes among GC patients in an ERAS setting. The study is conducted in the Surgical Gynecologic Department which includes a multidisciplinary clinic and a female surgical ward. The RCT will conform to the Consolidated Standards of Reporting Trials (CONSORT) Statement for reporting RCTs with two arms, comparing an intervention group to a control group. Following the RCT, the explanatory qualitative phase will be conducted among GC patients. Descriptive qualitative analysis will be employed to explore the perception of GC patients on the perioperative IMN nutrition intervention and to have a better understanding of the expectations of GC patients. Respondents will be recruited via purposive sampling. The study will be started on 1 September 2024 and end of the recruitment of this study on 1 September 2025.

### Study population

A total of 106 patients meeting the eligibility criteria will be enrolled in this study. Inclusion criteria encompass ambulatory adult Malaysian women scheduled for elective open surgery for GC (malignancy). Exclusion criteria involve individuals with allergies to soy or whey protein, diagnosed with chronic renal disease, ischemic heart disease, or diabetic mellitus, emergency surgery, minimally invasive surgery, vegan/vegetarian, and those participating in other interventional studies. The enrolment period spans from the clinic day until the postoperative day 30 of discharge (throughout operation management).

### Sample size calculation

Sample size estimation of RCT was calculated using two population means formula [17]. Prior data indicate that the mean handgrip changes of the control group was 0.7 (standard deviation = 0.4) and the mean of the intervention group was -1.4 (standard deviation = 4.8) [3]. Thus, a minimum sample size of 42 participants per group is required to reject the null hypothesis with a probability (power) of 0.8. The Type I error probability associated with this test is 0.05. The independent t-test statistic will be used to evaluate this null hypothesis. Considering an additional 20% dropout rate, the final sample size will be 53 participants per group.

### Recruitment

Participants are identified during their scheduled clinic appointment for gynecologist consultation on the treatment plan. Eligible patients (scheduled for elective open surgery), will be identified from the name list. Selection of patients will be done by assigned research team members then she/he will approach and inform with study procedures to all potential candidates (selected based on inclusion and exclusion criteria). The Patient Information Consent Form will be given to identify eligible participants who agreed to be recruited in the study. After consent, data will be recorded. On the same day of the multidisciplinary clinic, a comprehensive nutrition assessment (anthropometry, body composition, biochemical profile, and dietary assessment) and functional status assessment (handgrip strength, performance status, and physical activity level) will be carried out by a trained and credentialed dietitian.

### Randomization

The randomization process will assign participants to either the intervention group (I-ERAS) or the conventional group (CO) during consultation in multidisciplinary clinics. Since there will be two (2) comparison groups which will be intervention and control groups, a block size would be 4, where each block would contain 2 subjects from each group. This allocation follows the principles outlined in the CONSORT flow diagram, ensuring a rigorous and unbiased distribution of the participants into the respective groups. Block randomization, allocation concealment (opaque sealed envelopes), protocol adherence and intention-to-treat (ITT) analysis will be implemented to minimize the bias in the RCT. The Patient Information Consent Form will be provided to eligible participants who express their willingness to be part of the study. Participants are granted the option to take the consent form home for further discussion with family members if they desire additional input before making a final decision.

### Blinding

Given that this is an open-labeled RCT study, blinding will not be implemented. Participants, researchers, and healthcare providers will be aware of the assigned interventions, and the study will be conducted in an unmasked manner.

### Perioperative exercise

*Perioperative exercise*, including aerobic exercise like walking for 20–30 minutes, 3–5 times per week and deep breathing exercises, will be carried out to improve cardiovascular health and endurance lung function. Physiotherapist will review participant on preoperation, POD 1 and POD 2. Participant will ask to sit and ambulate on POD 1.

### Intervention group (I-ERAS)

Participants will attend a dietitian clinic to have anthropometry & dietary assessment during a multidisciplinary clinic. Participants will be given two servings of immunonutrition ONS (77g) (Valens Onthera^+^, PharmD Health Science, Malaysia) daily for five (5) days before tentative elective surgery. They will continue with protein-carbohydrate (CHO)-loading drinks in the evening before surgery and three hours before the operation. The I-ERAS group will receive 300ml of clear protein-CHO drink (contains 100g CHO; 12g protein) (evening drink) and 150ml clear protein-CHO drink (contains 50g CHO; 6g protein) (3 hours before operation drink) as a preoperative CHO loading regime. Participants will be fasted for solid food for 6 hours from the operation. Participants will be given 2 bottles of clear protein-CHO drink (400ml which contains 134g CHO; 16g protein) 4 hours post-surgery and will be reviewed by a dietitian. The on-duty staff nurse will keep an eye on the anesthetic risk of CHO loading and make sure the patient finishes certain drinks before the operation. When they tolerate at least 500 ml of clear fluids, they will be given a high protein high calories diet and continued 2 servings of IMN ONS (77g) (Valens Onthera^+^, PharmD Health Science, Malaysia) for post-operative seven (7) days. Patients will return the containers of the intervention product to monitor adherence. Strategies to improve and monitor the adherence to intervention protocols includes product container return.

### Control group (CO)

The participants will go to a dietitian clinic for anthropometry and dietary evaluation during a multidisciplinary clinic. Participants will continue their normal diet as tolerated before admission. Participants will fast for solids for 6 hours; they will receive 300ml of clear protein-CHO drink (contains 100g CHO; 12g protein) (evening drink) and 150ml clear protein-CHO drink (contains 50g CHO; 6g protein) (3 hours before operation drink) as a preoperative CHO loading regime. Participants will be fasted for solid food for 6 hours from the operation. Participants will be given 2 bottles of clear protein-CHO drink (400ml which contains 134g CHO; 16g protein) 4 hours post-surgery and will be reviewed by a dietitian. The on-duty staff nurse will keep an eye on the anesthetic risk of CHO loading and make sure the patient finishes certain drinks before the operation. After tolerating clear fluid, they will proceed to the postoperative high protein high calories diet. They will be prescribed 2 servings of polymeric formula daily only if unable to finish 75% of the diet served in the ward.

### Open-labeled RCT data collection procedure

Data collection will occur at various time points throughout the study, including baseline (on the day of the clinic appointment), the first (1^st^) day of the postoperative day (POD), POD 3 and POD 14. The study schedule of enrolment, interventions, and assessments is detailed (Table 1). Duplicate measurements and training of assessors will be done to promote data quality.

**Table 1:** Study schedule of enrolment, interventions, and assessments.

	STUDY PERIOD					
	Enrolment	Allocation	Post-allocation			Close-out
TIMEPOINT**	*Pre-operation*	0	*Pre-operation*	*POD1*	*POD3*	*POD14*
**ENROLMENT:**						
**Eligibility screen**	X					
**Informed consent**	X					
**Allocation**		X				
**INTERVENTIONS:**						
***I-ERAS***						
***CO***			X	X	X	X
**ASSESSMENTS:**						
Sociodemographic		X				
Clinical data		X				
Nutrition screening tool			X			
Anthropometry			X			
Body composition			X			X
Dietary intake			X	X	X	X
Biochemical data (albumin, CRP & full blood count)			X	X	X	X
Biochemical data (IgA, IgG, IgM, & interleukin-6)				X	X	X
Functional status			X			X
a) Hand grip strength			X			X
b) Performance status			X			X
Quality of life			X			X
Quality of sleep			X			X
Cognitive status, anxiety, and depression level			X			X
Post-operative outcomes			X			X
Post-operative complication						

CRP: C-Reactive Protein; Ig: Immunoglobulin; POD: postoperative day

### Study tools and parameters

#### Baseline characteristics

Participant characteristics, such as socio-demographic data (age, ethnicity, education level, and marital status), clinical characteristics (diagnosis, other comorbidities, and American Society of Anaesthesiologists (ASA) score, nutritional status [Patient Generated Subjective Global Assessment (PG-SGA)] score, weight change in the past 1-month, height, body composition and total daily energy protein intake), functional status (handgrip strength) and biochemical profile will be collected. Study tool and parameters are stated in Table 2.

**Table 2:** Study tool and parameters.

Variables	Tools/forms
Socio-Demographic a. Age b. Ethnic Group c. Education Level d. Marital status	Data collection form
Clinical data: Co-morbidities and Family history	Data collection form
Anthropometry, body composition and dietary intake	Data collection form
a) nutrition assessment tools	SGA, PG-SGA
b) body compositions (weight, muscle mass, fat free mass, fat mass, fat percentage)	Body Composition Monitor from Fresenius Medical Care
c) biochemical profile • Albumin • C-reactive protein • Full blood count • Immunoglobulin (IgA, IgG, IgM) • - Interleukin-6	Blood investigation
e) Dietary intake (daily total energy and protein intake)	24-hours dietary recall
Functional status a) Hand grips strength	Jamar hand dynamometer
b) Performance status	Karnofsky Performance Status
Quality of life	Questionnaire FAACT, 10-item Perceived Stress Scale (PSS-10) and EORTC QLQ-C30
Quality of sleep	Pittsburgh Sleep Quality Index (PSQI)
Cognitive status	FACT-Cognitive Function (Version 3)
Post-surgical outcome	Data collection form
a) Length of hospitalization	
b) Length of clear fluid toleration	
c) Length of diet toleration	
d) Length of bowel function return	
e) Postoperative complication	
• Postoperative nausea vomiting	
• Postoperative ileus	
• Wound debridement	
• Readmission 30-days after discharged	

SGA: Subjective Global Assessment; PG-SGA: Patient Generated Subjective Global Assessment; FAACT: The Functional Assessment of Anorexia/Cachexia Therapy; EORTC QLQ-C30: European Organization for Research and Treatment of Cancer Core Questionnaire.

#### Nutritional status

Nutritional status will be assessed by examining both within- and between-group differences in the intervention and control groups. Valid measurement tools, including the scheduled calibrated Body Composition Monitor from Fresenius Medical Care, will be employed. Anthropometric data, specifically height will be collected using the scheduled calibrated SECA ® 769 Height Measurement (up to 0.1cm) (SECA GmBH & Co., KG, Hamburg, Germany). Dietary intake will be assessed by 24-hour diet recall by a credentialed dietitian. Atlas of Food Exchanges and Portion Size [18], food models, and household measurements such as cups, spoons, and scoops were utilized to help participants judge the portion size of the foods they ate. Dietary intakes were analyzed by using Nutritionist Pro Dietary Software version 2.4 (San Bruno, CA, USA) [19]. A summary of the analysis included energy consumption in kilocalories (kcal) and protein intake in grams (g).

#### Biochemical profile

The biochemical profile will be the changes within- and between-group differences in the intervention and control groups. Blood investigation on the biochemical profile [albumin, c-reactive protein (CRP), full blood count (lymphocyte, neutrophil, monocytes, and platelet), immunoglobulin (Ig) A, IgG, IgM, and interleukin-6] will be ordered and collected by medical officers.

#### Functional status

Functional status will be assessed by examining both within- and between-group differences in the intervention and control groups. Handgrip strength will be measured using a Jamar hand dynamometer (Fred Sammons Inc, Illinois, USA). The final result will be based on the average of three successive attempts. The Performance status assessment, Karnofsky performance status (KPS) [20] will be used because it is widely used to quantify the functional status of cancer patients.

#### Quality of life, sleep quality and depression level

Quality of life (QoL) and stress level will be assessed for changes between the intervention and control groups. The QoL and stress level-related questionnaires FAACT, 10-item Perceived Stress Scale (PSS-10) and EORTC QLQ-C30 form while the sleep quality related questionnaire Pittsburgh Sleep Quality Index (PSQI) will be collected.

#### Clinical outcomes

Clinical outcomes will be evaluated based on differences between groups in various parameters including duration of hospital stays following surgery, ability to tolerate clear fluids, appetite, and restoration of bowel function. The duration between the completion of the surgical procedure to hospital discharge is referred to as the length of the postoperative hospital stay. The ability to tolerate clear fluid is defined as the duration from the end of the operation to the initial phase of tolerating clear fluids. The duration of food tolerance is defined as the time between the end of the operation and the resumption of regular meals. The duration of bowel function return is the time between the completion of the operation and the return of flatus. A gynecological surgeon will evaluate the clinical outcomes, and the charge nurse will document them on a data collection form (progress inward form). Appointment will be given to patients while discharged for post-discharged monitoring.

#### Explanatory qualitative study data collection

Before commencing data collection for the qualitative study, an interview guide in the form of a semi-structured questionnaire will be developed. The guide, characterized by open-ended questions, is designed to guide conversation and elicit comprehensive information from respondents [21].

Potential respondents will be selected from the participants of the phase I study. Subsequently, investigators will approach the potential respondents directly, providing a detailed explanation about the study and seeking consent upon discharge. The principal investigator will address any queries from potential respondents, ensuring a thorough understanding of the study. Once the potential respondents express a clear desire to participate and comprehend the study fully, they will be afforded adequate time to deliberate or consult with family members before signing the consent form and returning it to the investigator before the in-depth interview (IDI) session.

An IDI is a method that involves open-ended, discovery-oriented questions to obtain detailed and sensitive information about a topic from a respondent [22]. The investigator will explore in depth a respondent’s point of view, experiences, feelings, and perspectives. The principal investigator, along with a note-taker, will conduct the session in a private room (discussion room in the ward), guided by the interview guide. The session is expected to last up to one hour and audio recordings of the interview sessions will be taken.

Data collection is scheduled to span one year (September 2024 to September 2025) by a principal investigator who is proficient in both English and Malay language. Respondents will receive a comprehensive briefing on the data collection process including details about sampling and confidentiality. The investigator will record the audio data, and verification of all recorded data will be performed by both the principal investigator and co-investigator. Respondents may be contacted again if further information is required.

#### Withdrawal criteria

Participants may be withdrawn from the study if the investigator determines that continuing would be detrimental or pose a risk to the subject. Additionally, participants have the option to withdraw themselves from the study at any point without providing a specific reason. Withdrawn participants will not be replaced.

#### Adverse events and data safety monitoring

As of now, there are no known severe adverse effects associated with the study. If a participant experiences an adverse event during their enrollment, the medical record will be promptly updated. Participants facing adverse events will be directed to a doctor as soon as feasible, and the sponsor will cover the costs if it is a direct result of the research product or a medically necessary procedure for the study. The study doesn’t entail any additional medical care beyond standard procedures. In cases where adverse reactions arise from the consumption of study products, the staff nurse in charge will closely monitor the participants. Any unfavorable circumstances will be promptly reported to the medical officer in charge, who will initiate the necessary treatment. The study’s potential outcomes are expected to improve treatment results. The risk associated with participating in this study is considered minimal and is outweighed by the anticipated benefit, making the study worthy of encouragement. The sponsors will cover any medical expenses if any injuries arise as a direct result of taking part in the study. The cost of taking part in this study is not prorated.

#### Confidentiality, handling, and storage of data documents

Subject’s names will be kept anonymous and will be associated solely with a study identification number throughout the research. Subject data sheets will use the identification number instead of personal identifiers. All data will be entered into a computer that is password-protected. The audio recording will maintain anonymity, excluding any personal identifying information such as names or IC numbers during interviews. Audio recordings will solely serve transcription purposes and will neither be duplicated nor shared with another individual for alternative purposes. Following transcription, the audio recording will be disposed of securely. Upon completion of the study, data in the computer will be copied to CDs, and the data in the computer erased. CDs and any hardcopy data will be stored in a locked office of the investigators and maintained for a minimum of five years after the completion of the study. The CDs and data of the study will be destroyed after that period of storage (5 years). Respondents will not be allowed to view their study data, as the data will be consolidated into a database. Individuals involved in this study and the subjects’ medical care, qualified monitors and auditors, and governmental or regulatory authorities may inspect the study data and medical records, where appropriate and necessary.

#### Analysis plan

For phase I of the study (RCT), study data will be analyzed using the intention-to-treat (ITT) approach. The analyses will be performed using IBM SPSS Statistics for Windows (Version 22.0. Armonk, NY: IBM Corp.). Descriptive statistics will be applied for selected variables with results presented as frequencies and percentages for categorical data. Numerical Data which is normally distributed will be presented as mean and standard deviation while median and interquartile range will be presented for Numerical Data which is not normally distributed.

The continuous data that is normally distributed will be analysed using the Independent t-test, whereas data that is not normally distributed will be analysed using the Mann-Whitney test. Pearson’s Chi-square test for Independence will be used to study the association between categorical data and categorical data while Fisher’s exact test will be used when sample sizes are small and the expected frequencies in contingency tables are low (e.g., less than 5 in any cell). ITT analysis will be used to analyse the data to minimize bias. All probability values will be used two-sided and a level of significance of less than 0.05 (p-value < 0.05) will be considered as statistically significant.

For phase II (explanatory qualitative study), an inductive thematic analysis will be applied. All the recorded data will be transcribed before analysis. The thematic analysis will be carried out following a six-step process including familiarization, coding, generating themes, reviewing themes, defining and naming themes, and writing up [23]. NVivo software will be used to assist in organizing, coding, and analyzing qualitative data. Reflexive notes will maintained throughout the analysis to document thoughts and an audit trail will be preserved to ensure adherence to the procedure.

#### Ethical approval

The study will adhere to ethical principles outlined in the Declaration of Helsinki and the Malaysian Good Clinical Practice Guideline. All respondents will be given written consent before the study and will be briefed on the study protocol and procedure. Ethical approval was obtained from MREC with identity number NMRR ID 23-01663-0NI (IIR). The study protocol was registered in clinicaltrials.gov with identity number NCT06039306.

## Discussion

Maintaining overall good health, preventing infection, and supporting immunological surveillance against tumor cells necessitate optimal immune system functioning [24]. Nutrition plays a pivotal role in influencing immune system functions [25, 26]. The interplay of genetic makeup, cancer cell characteristics, and environmental factors collectively impact the immune system’s capacity to detect and combat cancer. The primary environmental influence of nutrition is the modulation of cell metabolism pathways by specific nutrients such as antioxidants, the immune system, and gut microbiota regulation, that operate systemically or locally within the malignant microenvironment [27].

IMN therapy targets immune defense mechanisms, preventing proinflammatory responses during the catabolic phase [25]. Various formulations and administration methods of IMN have been explored with varying success, primarily focusing on critical clinical outcomes (e.g., ventilator use, hospital stays, infection rates, and mortality) where immune response is vital [12, 24]. However, the impact of IMN within the ERAS setting on nutrition aspects (body composition, biochemical profile, and dietary intake), functional status, and quality of life remains unclear. The inflammation and bedrest during illness, surgeries, and hospital stays can contribute to rapid muscle loss, often associated with delayed recovery, slow wound healing, and reduced quality of life [27]. A comprehensive study on the effect of IMN on postoperative outcomes, nutrition, performance status, inflammation, and quality of life is yet to be explored.

In the ERAS setting, guidelines recommend shortening preoperative fasting with CHO loading and introducing early oral eating after surgery for improved postoperative results and reduced readmission rates [3, 5]. For women with GC, updated guidelines suggest CHO loading before surgery and early oral feeding after surgery [28]. Previous research demonstrated the positive effects of whey protein-infused CHO-loading drinks on surgical GC patients, preserving nutritional status, suppressing inflammatory responses, and reducing postoperative complications [2].

Muscle wasting is common in cancer patients and has been associated with undesirable outcomes, including higher mortality, dose-limiting toxicity, extended hospitalization, and surgical complications [29, 30]. Postoperative nutritional treatment, including ONS with HMB-enriched, is crucial for improving total energy and protein intake, preventing further muscle wasting in cancer and surgery-induced catabolism and pro-inflammatory states [30, 31]. The current study adds to the body of information on the role of HMB-enriched ONS in muscle preservation among GC patients in the ERAS context. The effectiveness of ONS depends on patient compliance, emphasizing the importance of patients’ understanding and preferences [32]. Implementing new protocols, such as perioperative IMN intervention under the ERAS setting, requires careful consideration due to potential adjustments in clinical intervention and management. The consistent and understandable information dissemination is essential to ensure multidisciplinary collaboration and adherence to the guidelines.

The perioperative IMN intervention in the ERAS setting aims to enhance patient-centered care, collaborative decision-making, and postoperative quality of life [5]. The study intervention could potentially become a standard model for perioperative nutritional intervention management, given its anticipated positive impact on patient outcomes and acceptance. The study contributes to the development of surgical oncology dietetic recommendations and provides a crucial tool for managing perioperative nutrition in surgical oncology patients.

In Malaysia, this study is the first qualitative explanation of the agreement between patient perceptions and evidence from trialed interventions to enhance adherence in GC. The study will utilize qualitative comparative analysis to investigate the level of agreement between qualitative analyses of patients’ perspectives and evidence from experimental interventions promoting adherence. Understanding patient perspectives is crucial for disease management, with better compliance associated with a focus on personal risk factors, the importance of adherence, and concise, relevant information on adherence.

The factor that determines the effectiveness of complicated interventions will be evaluated using the prospective value of employing qualitative research [16]. The intervention could be strengthened by gaining insight into how patients view the perioperative IMN intervention in the ERAS setting. Moreover, this is the pioneer study to examine the impact of the perioperative IMN intervention on postoperative outcomes among GC patients in the ERAS context. This study also will serve as the fundamental study to explore patients’ consciousness, perception, and acceptance of the related intervention approach. The present study will provide additional information to understand the impact of perioperative IMN intervention on postoperative clinical outcomes, nutritional outcomes (profile of body composition, nutrition intake, and biochemical outcomes (inflammatory marker and immunoglobulin)), functional outcomes (hand grip strength), performance status (Karnofsky Performance Status), quality of life, sleep quality; stress and anxiety level among GC patients in ERAS setting.

In conclusion, the study hypothesizes that the perioperative IMN intervention in an ERAS setting, combined with intensive nutritional intervention and monitoring, can preserve nutrition status, provide sufficient dietary intake for postoperative recovery, and improve outcomes after surgery. The lower immunological parameters during the postoperative healing phase may further benefit GC patients. Patients’ perceptions are expected to influence a better understanding of disease management and adherence to the intervention approach.

## Supporting information

S1 File SPIRIT-outcome 2022 checklist. (PDF)
